# Hepatitis C virus infection and the risk of intrahepatic cholangiocarcinoma and extrahepatic cholangiocarcinoma: evidence from a systematic review and meta-analysis of 16 case-control studies

**DOI:** 10.1186/s12957-015-0583-9

**Published:** 2015-04-23

**Authors:** Hao Li, Bin Hu, Zun-Qiang Zhou, Jiao Guan, Zheng-Yun Zhang, Guang-Wen Zhou

**Affiliations:** Department of Surgery, Medical Center of Digestive Disease, Shanghai Jiao Tong University Affiliated First People’s Hospital, No. 100, Haining Road, 200080 Shanghai, China; Department of Surgery, Shanghai Jiao Tong University Affiliated Sixth People’s Hospital, No. 600, Yishan Road, 200233 Shanghai, China

**Keywords:** HCV, Intrahepatic cholangiocarcinoma, Extrahepatic cholangiocarcinoma, Risk factor, Meta-analysis

## Abstract

**Background:**

Studies investigating the association between hepatitis C virus (HCV) infections and the occurrence of cholangiocarcinoma (CCA), especially intrahepatic cholangiocarcinoma (ICC), have shown inconsistent findings. Although previous meta-analyses referred to HCV and CCA, they mainly focused on ICC rather than CCA or extrahepatic cholangiocarcinoma (ECC). Since then, relevant new studies have been published on the association between HCV and ICC. Since the different anatomic locations of CCA have distinct epidemiologic features and different risk factors, it is necessary to evaluate the relationship between HCV infection and ICC, ECC, and CCA.

**Methods:**

Relevant studies were identified by searching PUBMED, EMBASE, and MEDLINE databases prior to 1 August 2013. Pooled risk estimates were calculated with random-effects models using STATA 11.0.

**Results:**

A total of 16 case-control studies were included in the final analysis. Pooled risk estimates showed a statistically significant increasing risk of CCA (odds ratio (OR) = 5.44, 95% CI, 2.72 to 10.89). The pooled risk estimate of ICC (OR = 3.38, 95% CI, 2.72 to 4.21) was higher than that of ECC (OR = 1.75, 95% CI, 1.00 to 3.05). In a subgroup analysis, the pooled risk estimate of ICC in studies from North America was obviously higher than in Asia (6.48 *versus* 2.01). The Begg funnel plot and Egger test showed no evidence of publication bias.

**Conclusions:**

HCV infection is associated with the increasing risk of CCA, especially ICC.

## Background

Cholangiocarcinoma (CCA) is a malignant neoplasm arising from the epithelial cells of the intrahepatic or extrahepatic bile ducts. Therefore, CCA can be classified as intrahepatic cholangiocarcinoma (ICC) and extrahepatic cholangiocarcinoma (ECC). CCA is the second most common primary hepatic malignancy, representing 10% to 25% of primary hepatic malignancies worldwide, accounting for 3% of all gastrointestinal tumors [1-3]. It has been shown that CCA is characterized by a low survival rate, with a median survival of less than 24 months [4].

Defining the risk factors for CCA benefits the search for better ways to prevent the occurrence of this disease. To date, it has been shown that hepatolithiasis, primary sclerosing cholangitis, liver flukes, biliary duct cysts, specific toxins (for example, the carcinogenic agent thorotrast), inflammatory bowel disease, and genetic polymorphisms are the major risk factors for CCA [5-8]. Although the association between CCA and viral hepatitis infections has been examined in several studies, the results remain controversial, especially for the hepatitis C virus (HCV) [7,9-15]. Several case-control studies have suggested that HCV infection plays a major role in CCA development [9-14]. In contrast, other studies have revealed no significant associations between CCA and HCV [7,15]. Therefore, in the present study, we performed a systematic review and meta-analysis to better evaluate the risk of CCA development in HCV-infected patients.

## Methods

### Literature search

Two independent reviewers searched PUBMED, EMBASE, and MEDLINE databases prior to 1 August 2013. The MeSH (Medical Subject Heading) search included headings that were all combinations of the hepatitis C virus such as: HCV, cholangiocarcinoma, intrahepatic, extrahepatic, bile duct cancer, and bile duct neoplasm. We also reviewed the reference lists of retrieved articles to search for more relevant studies. There were no language restrictions.

### Inclusion and exclusion criteria

Studies were eligible if they met the following criteria: (1) case-control studies; (2) exposure to HCV infection; (3) the outcome was CCA, ICC, or ECC incidence; and (4) providing risk estimates with 95% confidence interval (CI) or available information to calculate them. Abstracts, letters, reviews, case reports, studies lacking control groups, and studies that did not provide sufficient data to calculate risk estimates were excluded. Two investigators independently selected studies, and any discrepancies were resolved by consensus.

### Data extraction

For each eligible study, the following parameters were extracted independently by two researchers: (1) first author’s last name, date of study publication and the country where the study was conducted; (2) study design; (3) type of control; (4) sample size and age; (5) number of exposure in cases and controls; (6) risk estimate and 95% CI; and (7) adjustments and other information. The present meta-analysis included different measures of risk estimates (odds ratio, incidence rate ratio, standardized incidence ratio, and hazard ratio). In fact, the different measures yielded a similar risk estimate due to the relatively scarce occurrence of cholangiocarcinoma.

### Statistical analysis

A pooled risk estimate was calculated with a random-effects model, considering both intra-study and inter-study variances. Statistical heterogeneity was evaluated using the *Q* and *I*^2^ statistics. For the *Q* statistic, *P* < 0.10 was used as an indication of the existence of heterogeneity. Subgroup analysis was performed to explore potential heterogeneity among studies. Publication bias was assessed using the Begg funnel plot and the Egger test, and a *P* < 0.10 was considered as an indicator for publication bias [16,17]. All statistical analyses were conducted using STATA 11.0 for Windows (Stata, College Station, TX, USA).

## Results

### Search results

**Figure 1:**
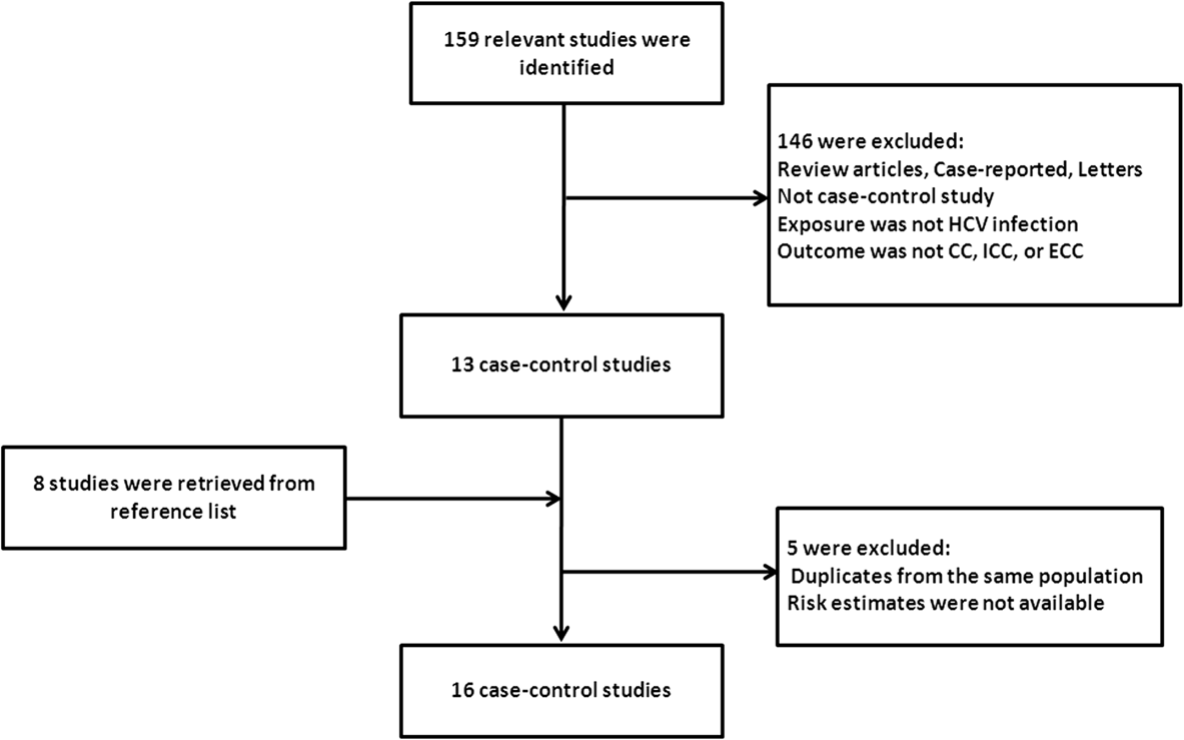
Flow chart of the selection and disposition of studies. A total of 159 relevant studies were identified during the initial search, and 16 studies remained after careful screening.

A total of 159 articles were identified through the literature search. Of these, 146 studies were excluded because they were either review articles, case reports, letters, or were not case-control studies. Studies were excluded when exposure was not an HCV infection and outcomes were not CCA, ICC, or ECC. The remaining 13 case-control studies, plus 8 studies from the reference lists, added 21 studies, of which 5 were excluded because of duplicates from the same population and risk estimates were not available. After strict screening, a total of 16 case-control studies were ultimately included in the present study [7,9-15,17-24] (Figure 1). The main features of these studies are shown in Table 1. We identified 16 case-control studies from 1991 to 2012. Four studies were from the USA [12-14,23], six from China [7,15,18,20,22,24], one from Taiwan [19], two from South Korea [9,17], one from Italy [10], one from Japan [11], and one from Thailand [21]. Data of a total of 3,658 patients with ICC and 1,280 with ECC were reported in these case-control studies. One study including 103 CCA cases did not classify ICC and ECC [21]. Among 5,044 cases, data of 140 patients with HCV infection were reported, whereas among 396,887 control subjects, data of a total of 1,879 subjects with HCV infection were reported. The diagnosis of CCA was confirmed by pathological examination. HCV infection was confirmed by laboratory tests. Controls recruited originated from hospital-based [7,9-11,13,15,17,19-22,24] or general population-based groups [12,14,18,23]. Control subjects [9-11,15,19,21] were selected from patients with non-liver-related cancer or other non-cancer disease and from healthy individuals at health screening centers of hospitals in four studies [13,17,20,22]. Qu *et al*. [24] selected participants with benign biliary disease with cholelithiasis or acute cholangitis as controls, whereas Liu *et al*. [7] selected patients with hepatolithiasis alone as controls. In 15 of the selected 16 studies, age-matched and sex-matched controls were used [7,9-15,17,19-24]. One study had two control groups [9]. Eight studies found a statistically significant positive association between HCV and ICC (odds ratio (OR) = 2.5 to 9.7) [9-14,19,23]. Four studies did not find a positive association between HCV and ICC [7,15,17,18], whereas two studies did not report the risk estimates [22,24].

**Table 1:** **Characteristics of 16 studies of HCV infection and the risk of cholangiocarcinoma**

**Study**	**Country**	**CCA type**	**Demographics**		**Case (n)**	**Control (n)**	**Sources**	**OR (95% CI)**	**Adjustments**
			**Age (Y)**	**Sex (% M)**					
Liu (2011) [7]	China	ICC	≧50	39	87	228	Hospital-based	0.87 (0.09 to 18.50)	NR
Shin (1996) [9]	Korea	ICC	59	73.2	41	406	Hospital-based	6.80 (2.30 to 20.30)	age, sex
Donato (20011) [10]	Italy	ICC	65	80.8	26	824	Hospital-based	9.70 (1.60 to 58.90)	sex, age, residence
Zhou (2008) [15]	China	ICC	53.2	66	312	438	Hospital-based	0.93 (0.28 to 3.10)	age, sex
Shaib (2005) [12]	United States	ICC	78.7	51.7	625	90,834	Population-based	6.10 (4.30 to 8.60)	age, sex, race, geographic location,
Shaib (2007) [13]	United States	ICC	59.8	55.4	83	236	Hospital-based	7.90 (1.30 to 84.50)	age, ethnicity, anti-HCV, HbsAg anti-HBc, alcohol consumption
		ECC	61.1	67.6	163			2.80 (0.30 to 35.10)	
Yamamoto (2004) [11]	Japan	ICC	64.6	58	50	205	Hospital-based	6.02 (1.51 to 24.1)	aspartate aminotransferase, blood transfusion, diabetes mellitus
Welzel (2007) [14]	United States	ICC	79	48	535	102,782	Population-based	4.40 (1.40 to 140)	age, race, geographic region
		ECC	78.7	51	549			1.50 (0.20 to 11.00)	
Lee (2008) [17]	South Korea	ICC	60.7	69.1	622	2,488	Hospital-based	1.00 (0.50 to 1.90)	age, sex
Hsing (2008) [18]	China	ECC	67	58.2	134	762	Population-based	0.80 (0.20 to 3.40)	education, smoking, BMI, diabetes, gallstones
Tao (2009) [20]	China	ICC	≧50	60.7	61	380	Hospital-based	6.30 (0.40 to 102.30)	NR
Lee (2009) [19]	Taiwan, Republic of China	ICC	61.5	63.1	160	160	Hospital-based	2.71 (1.16 to 6.32)	NR
Srivatanakul (2010) [21]	Thailand	CCA	NR	NR	103	103	Hospital-based	7/0 (1.44 to infinity)	anti-OV Ab
Welzel (2011) [23]	United States	ICC	76.4	47.5	743	195,953	Population-based	8.05 (5.08 to 12.75)	age, sex, race, geographic location
Cai (2011) [22]	China	ICC	56.6	62.0	313	608	Hospital-based	NR	NR
Qu (2012) [24]	China	ECC	63	63.9	305	480	Hospital-based	NR	NR

M, male; Y, year; CCA, cholangiocarcinoma; ECC, extrahepatic cholangiocarcinoma; ICC, intrahepatic cholangiocarcinoma; CI, confidence interval; OR, odds ratio; NR, not reported; BMI, body mass index; OV, opisthorchis viverrini; Ab, antibody.

### HCV infection and the risk of CCA

**Figure 2:**
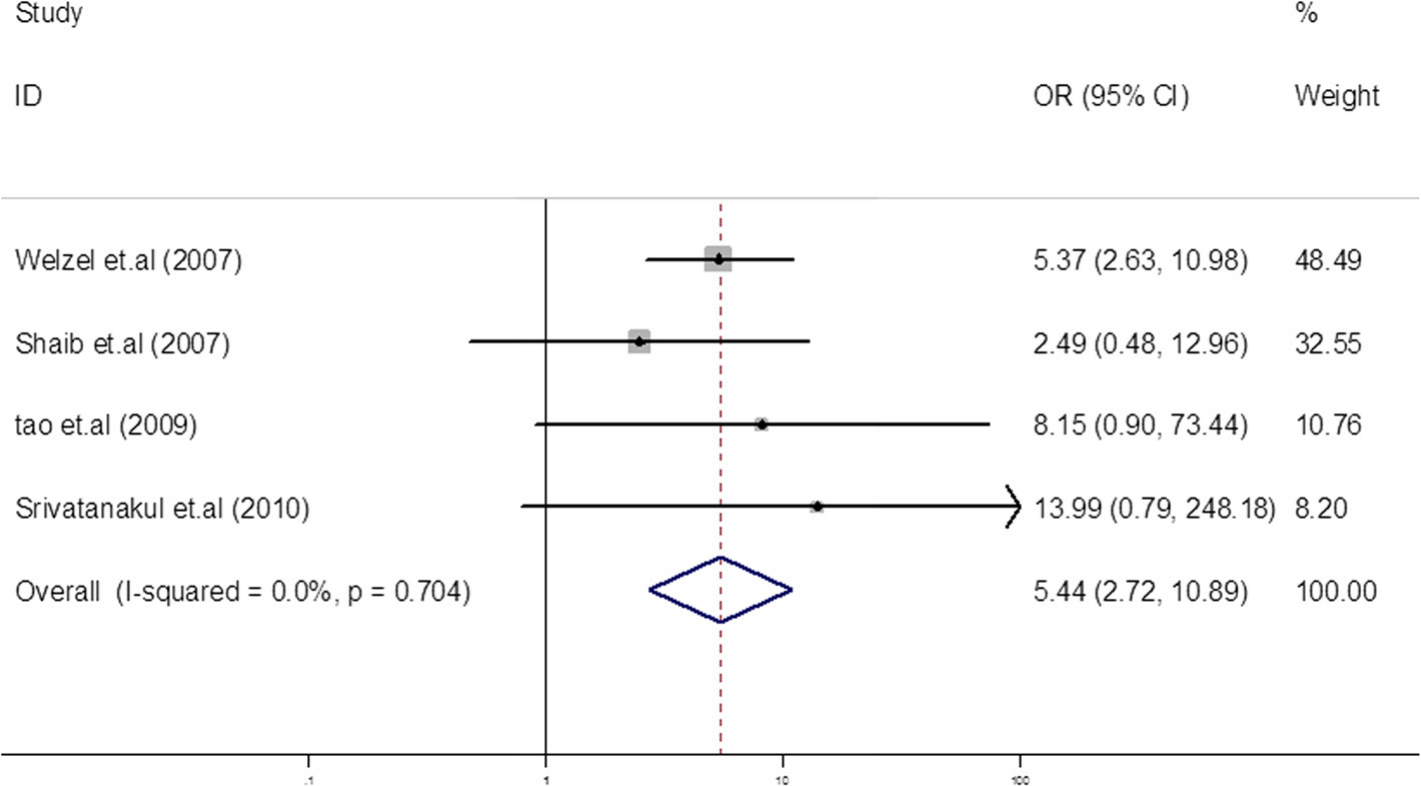
Forest plot of cholangiocarcinoma risk associated with HCV infection. A statistically significant positive association between HCV infection and CCA incidence was found (OR = 5.44, 95% CI, 2.72 to 10.89) in a random-effects model in the meta-analysis of four case-control studies.

We identified four case-control studies [13,14,20,21] reporting the association between HCV infection and the risk of CCA. All four studies found a positive correlation between HCV and the risk of CCA (OR = 2.49 to 13.99). In the present study, the meta-analysis of four case-control studies showed an OR of 5.44 (95% CI, 2.72 to 10.89) (Figure 2). The heterogeneity was not significant (*I*^2^ = 0.0%, *P* = 0.704).

### HCV infection and the risk of ICC

**Figure 3:**
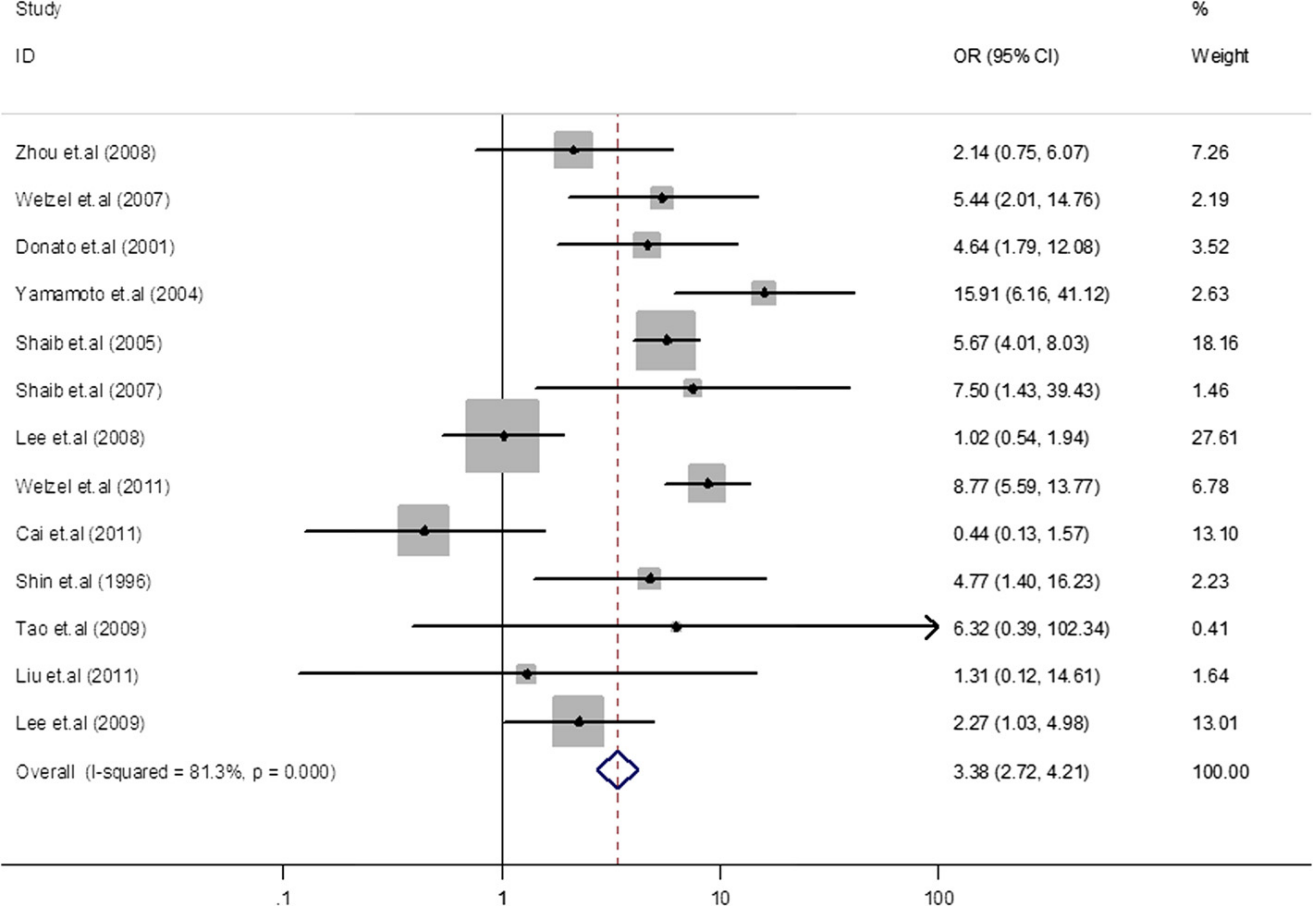
Forest plot of intrahepatic cholangiocarcinoma risk associated with HCV infection. A statistically significant positive association between HCV infection and ICC incidence was found (OR = 3.38, 95% CI, 2.72 to 4.21, *P* < 0.001) in a random-effects model in the meta-analysis of 13 case-control studies.

We found a statistically significant positive association between HCV infection and ICC incidence (OR = 3.38, 95% CI, 2.72 to 4.21, *P* < 0.001) in a random-effects model in the meta-analysis for 13 case-control studies [7,9-15,17,19,20,22,23], (Figure 3). In this meta-analysis, we found significant heterogeneity among the case-control studies (*Q* = 64.32, *I*^2^ = 81.3%, *P* < 0.001). In order to explore potential sources of heterogeneity, we conducted subgroup analyses by geographic region, sources, control groups, and adjusted covariates (Table 2). Among 13 case-control studies, 8 studies were from Asia [7,9,11,15,17,19,20,22], 4 from North America [12-14,23], and 1 from Europe [10]. The pooled OR of North American studies (OR = 6.48, 95% CI, 4.97 to 8.46) was higher than that of Asian studies (OR = 2.01, 95% CI, 1.44 to 2.79) and the European study (OR = 4.64, 95% CI, 1.79 to 12.08). The heterogeneity was significant in Asian studies (*I*^2^ = 77.30%, *P* < 0.001), but not in North American studies. The pooled ORs were 2.24 (95% CI, 1.65 to 3.05) for studies with hospitalized controls and 3.38 (95% CI, 2.72 to 4.21) with population-based controls. The heterogeneity was significant only in studies with hospitalized controls (*I*^2^ = 74.20%, *P* < 0.001).

**Table 2:** **Summary of risk estimates and 95% CI for the case-control studies**

**Study**	**Number of studies**	**OR (95% CI)**	**Tests for heterogeneity**		
			***Q***	***P*** **value**	***I*** ^**2**^ **(%)**
ICC	13				
Control group					
Hospital-based	10	2.24 (1.65 to 3.05)	34.93	<0.001	74.20
Population-based	3	3.38 (2.72 to 4.21)	2.43	0.297	17.60
Geographic region					
North America	4	6.48 (4.97 to 8.46)	2.44	0.486	0
Asia	8	2.01 (1.44 to 2.79)	30.83	<0.001	77.30
Europe	1	4.64 (1.79 to 12.08)	0		0
ECC	5				
Control group					
Hospital-based	3	1.66 (0.81 to 3.41)	5.80	0.055	65.50
Population-based	2	1.91 (0.81 to 4.50)	5.54	0.019	81.90
Geographic region					
North America	2	4.88 (1.88 to 12.65)	0.04	0.845	0
Asia	3	1.06 (0.52 to 2.19)	3.98	0.137	49.7%

OR, odds ratio; CI, confidence interval; ECC, extrahepatic cholangiocarcinoma; ICC, intrahepatic cholangiocarcinoma.

### HCV infection and the risk of ECC

**Figure 4:**
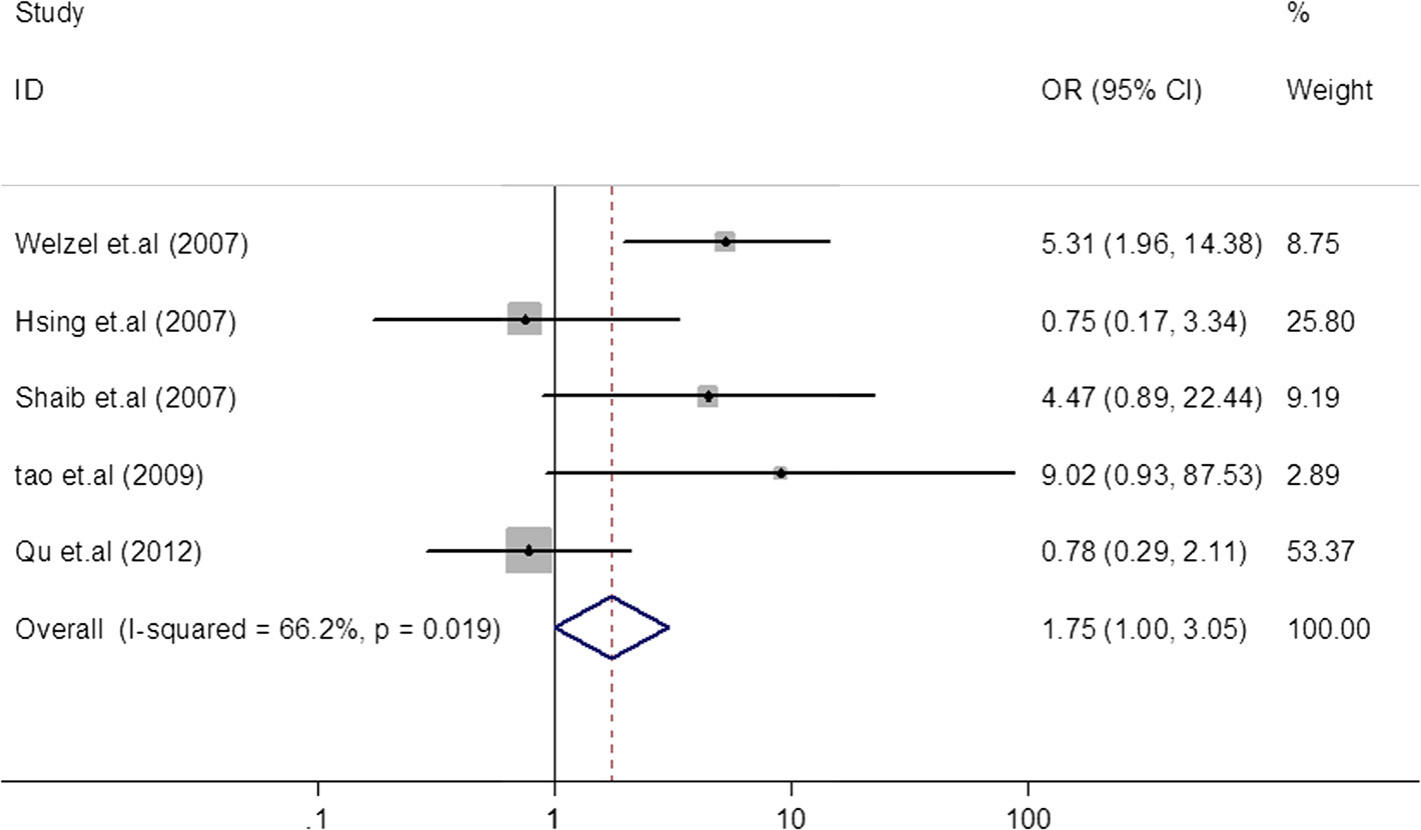
Forest plot of extrahepatic cholangiocarcinoma risk associated with HCV infection. The pooled risk estimate was 1.75 (95% CI, 1.00 to 3.051).

Five case-control studies reported results on HCV and ECC [13,14,18,20,24]. Among these studies, three studies [13,14,20] showed a statistically significant positive association between HCV and ECC (OR = 4.47 to 9.02), and two studies [18,24] showed no significant positive associations (OR = 0.75 to 0.78). In our meta-analysis of these studies, the pooled risk estimate was 1.75 (95% CI, 1.00 to 3.05) in a random-effects model (Figure 4).

In the subgroup analysis (Table 2), three studies were from Asia, and two from North America. The pooled OR of North American studies (OR = 4.88, 95% CI, 1.88 to 12.65) was higher than that of Asian studies (OR = 1.06, 95% CI, 0.52 to 2.19). No significant heterogeneity was shown in any of the subgroups. The pooled ORs were 1.66 (95% CI, 0.81 to 3.41) for studies with hospitalized controls and 1.91 (95% CI, 0.81 to 4.50) with population-based controls. Significant heterogeneity was found between studies with hospitalized controls and studies with population-based controls.

### Publication bias

**Figure 5:**
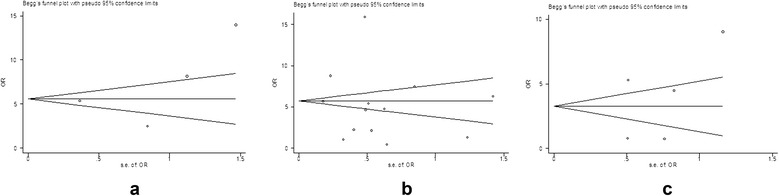
Funnel plot of four studies. **(a)** No publication bias was found between HCV infection and CCA risk using Begg’s adjusted rank correlation test (*P* = 0.308) and Egger’s regression asymmetry test (*P* = 0.485). Funnel plot of 13 studies; **(b)** no publication bias was found between HCV infection and ICC risk using Begg’s adjusted rank correlation test (*P* = 0.951) and Egger’s regression asymmetry test (*P* = 0.606). Funnel plot of 5 studies; **(c)** no publication bias was found between HCV infection and ECC risk using Begg’s adjusted rank correlation test (*P* = 0.221) and Egger’s regression asymmetry test (*P* = 0.495).

Funnel plots showed no publication bias in studies concerning CCA, ICC, and ECC (Figure 5). *P* values for Begg’s adjusted rank correlation test and Egger’s regression asymmetry test were positive (*P* > 0.05), suggesting that publication bias had little effect on summary estimates.

## Discussion

The global prevalence of CCA has shown a rising trend in recent years, and the incidence of HCV infection is also found to be elevated [2,25-28]. Although many researchers have focused on the association between HCV infection and the risk of CCA, the findings have varied due to differences between the studies in geographic regions, study design, sample size, and controls. Also, in previous meta-analyses, adjusted risk estimates were used rather than the unadjusted risk estimates used in the present study. Regarding the potential confounding factors for CCA, summary risk estimates are more precise and believable when using findings from adjusted models. Although previous studies referred to HCV and CCA, they mainly focused on HBV rather than HCV, and ICC rather than ECC or CCA [29,30]. Further, just as different anatomic locations of CCA have distinct epidemiologic features and different risk factors [13], ICC and ECC also have many differences. The present study has compensated for these deficiencies by focusing on HCV and CCA, including ICC and ECC. Moreover, the 16 case-control studies included in our meta-analysis exceed the number of studies included in previous meta-analysis. Therefore, results and conclusions of our data analysis are more reliable than previously published meta-analyses. The present meta-analysis was conducted to evaluate the relationship between HCV infection and the risk of ICC, ECC, and CCA and was not confined to associations between HCV and ICC.

The present study found that patients with an HCV infection have an approximately 5.44-fold increased risk of CCA compared to individuals with a non-HCV infection. In the study by Srivatanakul *et al*. [21], the OR for CCA was 4.00. These results indicate that HCV is associated with CCA incidence. To gain insight into the association between HCV and CCA, we classified CCA into ICC and ECC. The pooled OR of ICC (OR = 3.38, 95% CI, 2.72 to 4.21) was higher than that of ECC (OR = 1.75, 95% CI, 1.00 to 3.05). This can probably be explained by the different anatomic locations of CCA, which were associated with different risk factors for CCA. From these results, we concluded that ICC was probably more susceptible to HCV infection than ECC. In subgroup analysis of HCV and the risk of ICC in different geographic regions, the pooled OR was obviously higher in studies from North America than those from Asia (6.48 *versus* 2.01). This phenomenon was also seen in the subgroup analysis of HCV and the risk of ECC (4.88 *versus* 1.06) in the different geographic regions. This might be explained by the fact that hepatitis C was the most prevalent form of viral hepatitis infection in Western countries compared to Asian countries. As many studies have reported, ICC incidence is rising in Western countries, which may reflect that regional differences have led to different risk factors and epidemiological factors associated with ICC [2,29,31,32]. In subgroup analyses of controls, studies using hospital-based controls and those using population-based controls had similar risk estimates for ICC (2.24 *versus* 3.38) and ECC (1.66 *versus* 1.91).

The mechanism of CCA development in patients with HCV infection has not yet been clarified in detail. One study suggests that the presence of HCV in the bile duct epithelium indicates that HCV infection can cause chronic inflammation of the bile duct epithelium [33]. Long-term expression of HCV oncoproteins may be involved in the tumourigenic process leading to tumor formation [34]. Another study reported that HCV core variants could alleviate TGF-beta cytostatic responses and increase TGF-beta-mediated epithelial-to-mesenchymal transition (EMT), thereby promoting cell invasion and metastasis [35]. Likewise, Chen *et al*. [36] found that HCV core protein promoted the cellular proliferation of hilar cholangiocarcinoma cells and inhibited apoptosis. Clearly, based on such results, HCV can lead to hepatocellular carcinoma (HCC). Moreover, some researchers have suggested that hepatocytes and cholangiocytes have the same progenitor cells, which are hepatic progenitor cells, especially in ICC and HCC [37]. Therefore, it is possible that HCV induces carcinogenesis in cholangiocytes and hepatocytes by the same mechanism. Recently, HCV RNA has been detected in ICC specimens, which further indicates the potential role of HCV infection in the pathogenesis of ICC [36,38,39]. Since HCV RNA or its components can be detected in bile duct epithelium [40,41], our results demonstrate a slight positive association between HCV infection and ECC. HCV infection can increase the risk of ECC by 1.75%. This conclusion is consistent with studies by both Welzel *et al*. [12] and Shaib *et al*. [14]. However, Qu *et al*. [24] found that the seroprevalence of anti-HCV was 4.3% in ECC patients and 5.6% in patients with benign biliary disease but without significant differences. HCV infection has been shown to be associated with ICC and its increasing incidence, but not with ECC or its incidence [14]. Similarly, Matsumoto *et al*. [42] also found that HCV-Ab was 20% in ICC patients and 7.4% in ECC patients [42]. Results of both of these studies explain that HCV is the major risk factor for ICC but not for ECC.

This meta-analysis has several limitations that should be considered when interpreting the results. First, conducting randomized clinical trials was not feasible for an observational study of HCV and the risk of CCA. All studies included in this meta-analysis were case-control studies that might introduce selection and recall biases, which may possibly distort the true associations between HCV and CCA. Second, seropositivity for anti-HCV was used as the sole marker of HCV infection, and the definition of HCV infection was not stated in most studies, therefore a common definition may be lacking. Occult HCV infection might also play a role in the development of CCA. Thus, for these reasons, the HCV effect may be underestimated. Third, the diagnostic misclassification of cholangiocarcinoma (that is, hilar cholangiocarcinoma classification as intrahepatic or extrahepatic tumors) might result in a mismatch of true ICC or ECC.

## Conclusions

The results of the present study reveal that HCV is associated with the significantly increasing risk of CCA, including ICC and ECC, especially ICC. Further studies are warranted to gain a better understanding of the precise mechanism between HCV and CCA.

## Abbreviations

HCV: hepatitis C virus
CCA: cholangiocarcinoma
ICC: intrahepatic cholangiocarcinoma
ECC: extrahepatic cholangiocarcinoma
CI: confidence interval
OR: odds ratio
